# Predictive Value of Cerebrospinal Fluid Biomarkers for Tap Test Responsiveness in Patients With Suspected Idiopathic Normal Pressure Hydrocephalus

**DOI:** 10.3389/fnagi.2021.665878

**Published:** 2021-05-20

**Authors:** Rongrong Hua, Chunyan Liu, Xing Liu, Jinwu Zhu, Jie Zhang, Lidong Wang, Zhe Shi, Jian Li, Shuangyan Kong, Chenhui Yang, Nan Liu, Lijuan Liu, Jie Sun, Qiong Yang, Yubing Wu, Ying Zhou, Yanfeng Li, Yan Xing

**Affiliations:** ^1^Department of Neurology, Aviation General Hospital, Beijing, China; ^2^Aviation Medical Engineering Center of Aviation General Hospital, Beijing, China; ^3^Department of Neurology, Peking Union Medical College Hospital, Beijing, China

**Keywords:** idiopathic normal pressure hydrocephalus, cerebrospinal fluid biomarkers, tap test, tau, Aβ

## Abstract

**Background:** The value of cerebrospinal fluid (CSF) biomarkers for assessing idiopathic normal pressure hydrocephalus (iNPH) must be determined. This prospective study aimed to reveal the correlation between CSF biomarkers and clinical symptoms of iNPH and the predictive value of these biomarkers for tap test responsiveness.

**Methods:** Thirty-nine patients with suspected iNPH were recruited, contributed qualified CSF, and underwent a tap test and unified pre- and post-test evaluations of the neurological function.

**Results:** The analysis of biomarkers from the patients’ CSF showed decreased levels of tau and its phosphorylated form, especially in the tap test (+) group. The responsiveness of the tap test was also related to the number of combined symptoms (*p* < 0.01), and a correlation was found between the end pressure or pressure difference in CSF and tap test responsiveness (*p* < 0.05). The results of the binary logistic regression analysis showed that *P* (tap test responsiveness) = 1/1 + e^∧^ − (−5.505 + 55.314 * ratio of p/*T*-tau − 1.586 * numbers of combined symptoms). The combined indicators (−5.505 + 0.553 * percentage of p/*T*-tau − 1.586 * numbers of combined symptoms) resulted in the highest sensitivity and specificity of 94.12% and 72.73%, respectively.

**Conclusions:** CSF biomarkers may be assessed to judge tap test responsiveness, which is beneficial for the feasibility of a clinical application.

## Introduction

Idiopathic normal pressure hydrocephalus (iNPH) is a critical brain disorder without a clear cause that occurs in adults and is accompanied by excess cerebrospinal fluid (CSF) accumulation in the ventricular system, resulting in gait dysfunction, a frontal-subcortical pattern of cognitive impairment, or urinary urge incontinence with insidious onset (Williams and Malm, 2016; Liew et al., 2019). The pathophysiology of iNPH is most likely multifactorial and mediated by a disturbance in CSF dynamics (Graff-Radford and Jones, 2019), although clinicians are more likely to combine key clinical symptoms and imaging findings to diagnose iNPH. Shunt placement is recommended as an effective treatment for patients with iNPH, although large double-blind studies are still necessary (Halperin et al., 2015). Approximately 60–80% of patients may benefit from shunt surgery (Toma et al., 2013). Unfortunately, there is no reliable biomarker for iNPH to assist in the selection of patients who would benefit from shunt surgery.

Researchers recently reported data indicating that the presence of amyloid beta 1–42 (Aβ1–42), the pathological hallmark of Alzheimer disease (AD) in cortical biopsies obtained at the time of shunt placement, is associated with a poorer response to shunting in patients with iNPH (Abu Hamdeh et al., 2018). Although the quantification of CSF biomarkers, such as Aβ1–42, total tau (*T*-tau), and phosphorylated tau (*p*-tau) proteins, has been incorporated into standard diagnostic guidelines for degenerative diseases such as AD, potential biomarkers for the diagnosis of hydrocephalus and responsiveness to receiving a shunt must also be explored. In addition, the neurodegenerative markers *T*-tau, ^181^*p*-tau, and Aβ1–42 successfully differentiate between AD and iNPH and therefore may be candidate biomarkers for determining the prognosis and shunt response of patients with iNPH (Jingami et al., 2015a). However, the details remain unclear. The aim of this study was to explore the related factors of the tap test and the predictive value of CSF *T*-tau, ^181^*p*-tau, and Aβ1–42 levels for shunt responsiveness.

## Materials and Methods

### Patients

The study protocol was approved by the Ethics Committee of Aviation General Hospital. All the methods were performed in accordance with the relevant guidelines and regulations (Relkin et al., 2005; Mori et al., 2012). The patients were recruited consecutively from the Neurology Department at Aviation General Hospital, China Medical University from January 1, 2017, to August 1, 2019. The inclusion criteria were age >50 years and iNPH diagnosed according to the guideline (Mori et al., 2012), which includes: (1) dementia, gait failure, or urinary incontinence in the absence of a preceding disorder, including meningitis, aqueduct stenosis, subarachnoid hemorrhage, and other pathology, and (2) an Evans index >0.3 according to CT or magnetic resonance imaging (MRI). The available medical information was obtained from medical records and treating physicians. Patients with pure iNPH (*n* = 42) were recruited, and a copathology (AD, PD or, vascular comorbidity) was excluded by PET, MRI, and tremor analyses. All the patients underwent standard screening at baseline, including physical and neurological examinations, MRI, and laboratory tests. Diagnoses were made by a multidisciplinary team who achieved a consensus without knowledge of the CSF results. Informed consent was obtained from all the participants and/or their legal guardians. All the subjects provided written informed consent for the use of clinical data for research purposes.

### Tap Test and CSF Collection

Lumbar puncture should preferably be performed in the morning. After a routine examination excluded surgical contraindications, an intervertebral lumbar puncture was performed at L3–4 or L4–5 under local infiltration anesthesia. After confirming that the needle had entered the subarachnoid space, the initial pressure of CSF was determined. Then, the CSF (total volume = 30 ml) was collected and placed in two 15 ml polypropylene centrifuge tubes (430791, Corning, New York, USA), which were immediately centrifuged ±3,700 *g* for 10 min at 4°C to exclude insoluble materials. Then, the fluid was dispensed into 1 ml polypropylene tubes and stored at −80°C. After measuring the end pressure of CSF and removing the lumbar puncture needle, the tap test was complete. The laboratory staff was blinded to the clinical information of each subject.

### CSF Analysis

CSF was sampled to measure *T*-tau, *p*-tau, and Aβ1–42 levels during the reservoir tap test. The CSF samples were collected under sterile conditions into two 15 ml centrifuge tubes. The CSF sample collection and storage methods were all performed in accordance with the consensus guidelines for CSF biobanking. The samples were sent to measure the concentrations of *T*-tau (INNOTEST hTAU Ag ELISA kit, Fujirebio Europe N.V., Belgium), ^181^*p*-tau (INNOTEST PHOSPHO-Tau (181p) ELISA kit, Fujirebio Europe N.V., Belgium), and Aβ–42 [INNOTEST β-AMYLOID (1–42) ELISA kit, Fujirebio Europe N.V., Belgium] and total proteins for enzyme-linked immunosorbent assays (ELISAs). A technician who was blinded to the clinical results prospectively recorded the levels of *T*-tau, ^181^*p*-tau, and Aβ1–42. The longitudinal stability of the measurements was ascertained using an elaborate program of internal quality control (QC) samples. Intra-and inter-assay coefficients of variation were 1.37 and 5.8% for Aβ1–42, 2.51 and 1.46% for *T*-tau, 3.36 and 1.52% for *p-tau*, respectively.

### Standard Reference: Clinical Outcome

The clinical outcome groups “tap test-positive” or “tap test-negative” provided the reference standard. Outcome measures were recorded prospectively and analyzed retrospectively. As described in a previous study (Ishikawa et al., 2012), three main objective outcome measures were analyzed: (1) a more than three-point improvement in the Mini-Mental State Examination (MMSE); (2) a minimum of 20% improvement in either time in seconds or number of steps or a 10% improvement in both the timed 10 m walking test; and (3) a more than 10% improvement in time on the up-and-go test (TUG). The assessments were performed by personnel who were blinded to the index test result. For the outcome analysis, a “positive (+) tap test” reflected a better outcome in at least one of the three objective measures after the tap test, in addition to reported subjective improvement. A deterioration or no change in any one of these clinical elements resulted in an overall outcome of “negative (−) tap test.” All the outcome data were processed using an anonymous database.

### Statistical Analysis

Normally distributed data are presented as the means ± standard deviations. Continuous variables with a normal distribution from two groups were compared using an independent *t*-test. Continuous variables with a normal distribution from more than two groups were compared using one-way ANOVA. Data with a nonnormal distribution are described as the medians (25th and 75th percentiles). The Mann-Whitney U test or Kruskal-Wallis H test was used to compare data with a nonnormal distribution from different groups. The conditional count data were compared using a chi-square test. Spearman’s or Pearson’s correlation analysis was applied to examine the correlations. A binary logistic regression analysis was used to select and clarify the contribution of the related factors to the tap test responsiveness. ROC curves were analyzed, and a cutoff value was selected. A *p* value of 0.05 was defined as the threshold of statistical significance in each test.

## Results

### Study Profile

**Figure 1:**
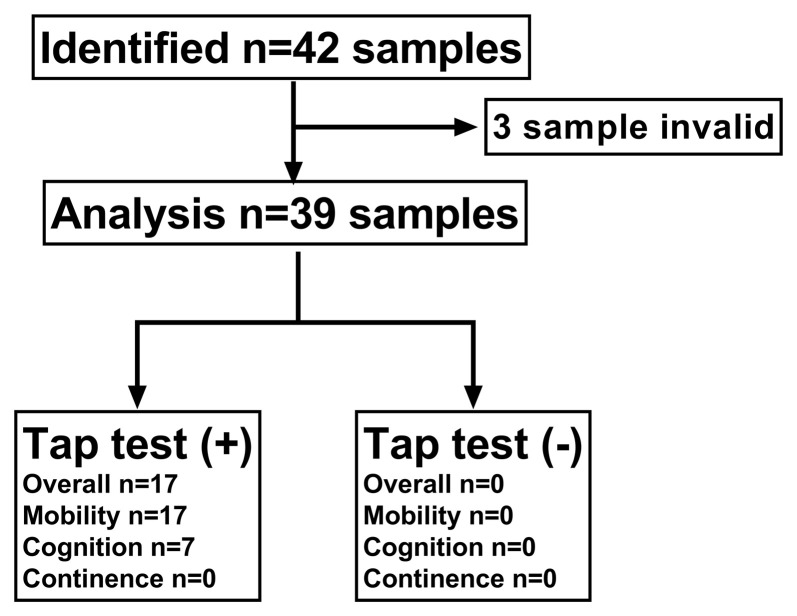
Flow chart of participating patients. Thirty-nine patients with suspected idiopathic normal pressure hydrocephalus (iNPH) were included in the final predictive value analysis. Secondary samples that underwent a tap test (+) (*n* = 17) or (−) (*n* = 22) were included in the longitudinal analysis of biomarkers in lumbar cerebrospinal fluid (CSF).

From January 1, 2017, to August 1, 2019, 39 CSF samples from 42 patients with suspected iNPH were analyzed: six women and 33 men with a mean age 71.44 ± 8.10 (range 52–85) years. Figure 1 outlines the study profile.

### The Levels of Lumbar Aβ1–42, *T*-tau, and, *p*-tau and Their Relationship With the Tap Test Response or Numbers of Combined Symptoms

**Figure 2:**
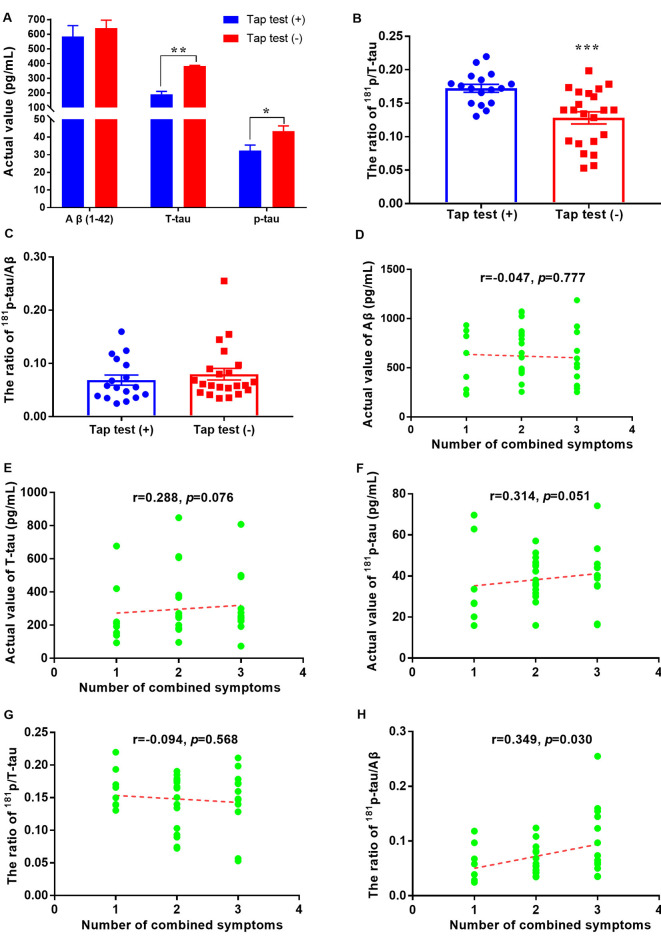
Aβ1–42 and tau levels and their relationships with tap test responsiveness or combined symptoms in patients with iNPH. **(A)** Comparison of CSF levels of Aβ1–42, *T*-tau, and *p*-tau between the tap test (+) and (−) groups (* *p* < 0.05 and **p < 0.01 compared with the corresponding group). **(B)** Comparison of the ratio of ^181^p/*T*-tau in the tap test (+) and (−) groups (***p < 0.001 compared with the corresponding group). **(C)** Comparison of the ratio of ^181^*p*-tau/Aβ1–42 between the tap test (+) and (−) groups. **(D–H)** Correlation analyses between the actual levels of Aβ1–42, *T*-tau, *p*-tau, p/*T*-tau, and *p*-tau/Aβ1–42 with the numbers of combined symptoms.

According to previous studies, patients with iNPH tend to exhibit low CSF *T*-tau levels, which may be a good predictor of the postoperative outcome. Similarly, in our study, the levels of tau and its phosphorylated form were significantly decreased, especially in the tap test (+) group (*p* < 0.01 and *p* < 0.05), although the levels of Aβ1–42 did not change significantly (Figure 2A). The levels of Aβ1–42 in the tap test (+) and (−) groups were 584.86 ± 303.55 pg/ml (*n* = 17) and 641.36 ± 255.38 pg/ml, respectively (*n* = 22; Figure 2A). The levels of *T*-tau in the tap test (+) and (−) groups were 191.21 ± 80.52 pg/ml (*n* = 17) and 382.88 ± 25.35 pg/ml, respectively (*n* = 22; Figure 2A). The levels of *p*-tau in the tap test (+) and (−) groups were 32.38 ± 12.63 pg/ml (*n* = 17) and 43.38 ± 14.02 pg/ml, respectively (*n* = 22; Figure 2A). In contrast, the ratio of p/*T*-tau in the tap test (+) group was 17.23 ± 0.59%, which was higher than that in the tap test (−) group (12.81 ± 0.90%) (*p* < 0.001; Figure 2B). The results suggested that the magnitude of the reduction in *p*-tau levels was significantly lower than that of *T*-tau levels, resulting in a relatively higher p/*T*-tau ratio. In addition, the ratio of *p*-tau/Aβ1–42 was 6.87 ± 0.95% and 7.97 ± 1.09% in the tap test (+) and (−) groups (12.81 ± 0.90%), respectively (*p* > 0.05; Figure 2C).

A correlation analysis was performed to analyze the relationship between CSF biomarkers and the number of combined symptoms of iNPH patients. Although the *p* values for the correlation analysis between the actual levels of Aβ1–42, *T*-tau, *p*-tau, or *p*/*T*-tau with the number of combined symptoms were 0.777, 0.076, 0.051, and 0.568, respectively, a significant difference in the number of combined symptoms that correlated with the *p*-tau/Aβ1–42 ratio was found (*p* = 0.030; Figures 2D–H).

**Figure 3:**
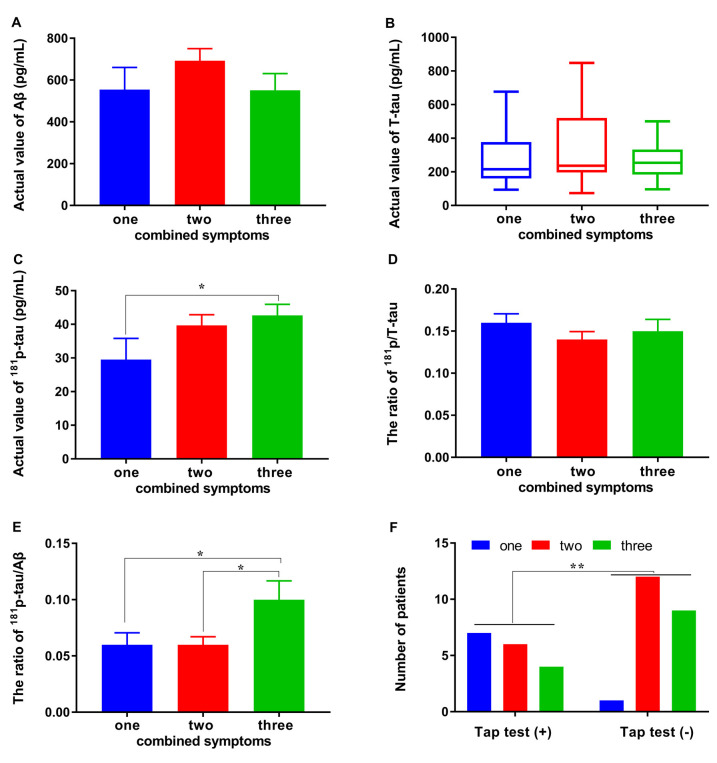
Differences in CSF biomarkers and tap test responsiveness between subgroups with different numbers of combined symptoms. **(A–E)** Differences in the levels of Aβ1–42, *T*-tau, ^181^p/*T*-tau, and ^181^*p*-tau/Aβ1–42 between the subgroups with different numbers of combined symptoms. **(F)** The relationship of the tap test responsiveness with number of combined symptoms (*p < 0.05 and **p < 0.01 compared with the corresponding group. The data in **(B)** are presented as the medians with interquartile ranges).

A subgroup analysis was used to determine the accurate significant difference of each CSF biomarker in groups with one/two/three combined symptoms. Unfortunately, no difference was observed between the subgroups with different numbers of combined symptoms and Aβ1–42, *T*-tau, and p/*T*-tau (*p* > 0.05, *p* > 0.05 and *p* > 0.05; Figures 3A,B,D). The levels of Aβ1–42 in the subgroups with one/two/three combined symptoms were: 554.31 ± 300.82, 692.15 ± 247.35, and 550.73 ± 290.23 pg/ml, respectively (Figure 3A). The levels of *T*-tau in the subgroups with one/two/three combined symptoms were: 215.65 (160.76, 377.24), 236.12 (196.09, 520.71), and 253.62 (183.47, 333.22) pg/ml, respectively (Figure 3B). The p/*T*-tau ratios in the subgroups with one/two/three combined symptoms were: 0.16 ± 0.03, 0.14 ± 0.04, and 0.15 ± 0.05, respectively (Figure 3D). However, the levels of *p*-tau in the subgroups with one/two/three combined symptoms were: 29.50 ± 17.89, 39.70 ± 13.34, and 42.65 ± 11.81 pg/ml, respectively, and the difference between the groups with one and three combined symptoms was significant (*p* < 0.05; Figure 3C). The *p*-tau/Aβ1–42 ratios in the subgroups with one/two/three combined symptoms were: 0.06 ± 0.03, 0.06 ± 0.03, and 0.10 ± 0.06, respectively, and the differences between the groups with one/two and three combined symptoms were significant (*p* < 0.05 and *p* < 0.05; Figure 3E). In addition, the responsiveness to the tap test was also related to the number of combined symptoms (Figure 3F). The numbers of patients with one/two/three combined symptoms in the tap test (+) and (−) groups were 7/6/4 and 1/12/9, respectively. Significantly more combined symptoms were observed for the patients in the tap test (−) group (*p* < 0.01).

### The Distribution of Combined Symptoms and Its Correlated Factors

**Figure 4:**
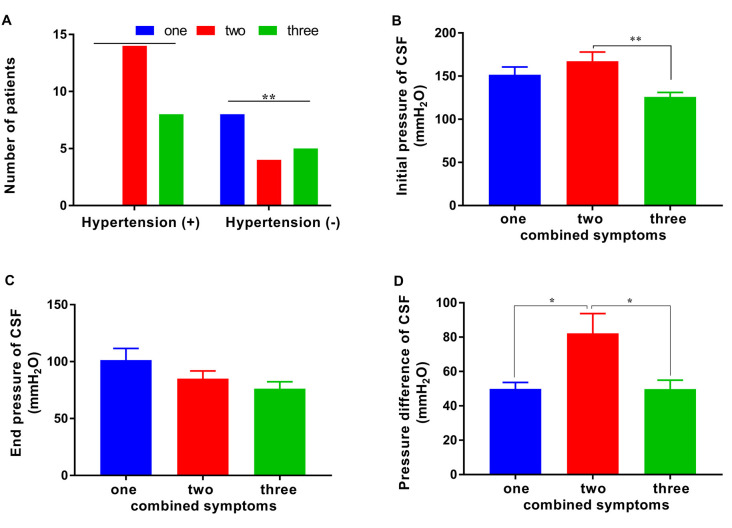
The distribution of combined symptoms and the correlated factors. **(A)** The distribution of hypertension in patients with one/two/three combined symptoms. **(B–D)** The CSF initial pressure, end pressure and, pressure difference in patients with one/two/three combined symptoms (*p < 0.05 and **p < 0.01 compared with the corresponding group).

We showed that patients with a fewer number of combined symptoms may have a positive response to the tap test. Further analysis revealed that the prevalence of hypertension, initial pressure, and pressure difference in CSF were also related to the number of combined symptoms. Significant differences in the distribution of combined symptoms of patients with or without hypertension were observed (*p* < 0.01). The number of patients with hypertension presenting one/two/three combined symptoms was 0/14/8 compared with 8/4/5 in the nonhypertensive patients (Figure 4A). This finding suggested that patients with hypertension would have more symptoms of iNPH than those without hypertension. The initial pressure of CSF in the patients with two symptoms of iNPH was 167.22 ± 45.45 mmH_2_O, a value that was significantly higher than that in the patients with three symptoms (125.92 ± 18.67 mmH_2_O) and similar to that in the patients with one symptom (151.50 ± 25.51 mmH_2_O; Figure 4B). The pressure difference in the patients with two symptoms of iNPH was 82.22 ± 48.63 mmH_2_O, a value that was significantly higher than that in the patients with one symptom (49.88 ± 10.63 mmH_2_O) or three symptoms (49.77 ± 18.75 mmH_2_O; *p* < 0.05 and *p* < 0.05), although the end pressure of CSF was not significantly different in the patients with iNPH presenting different numbers of combined symptoms (Figures 4C,D).

### The Responsiveness to the Tap Test and Its Correlated Factors

**Figure 5:**
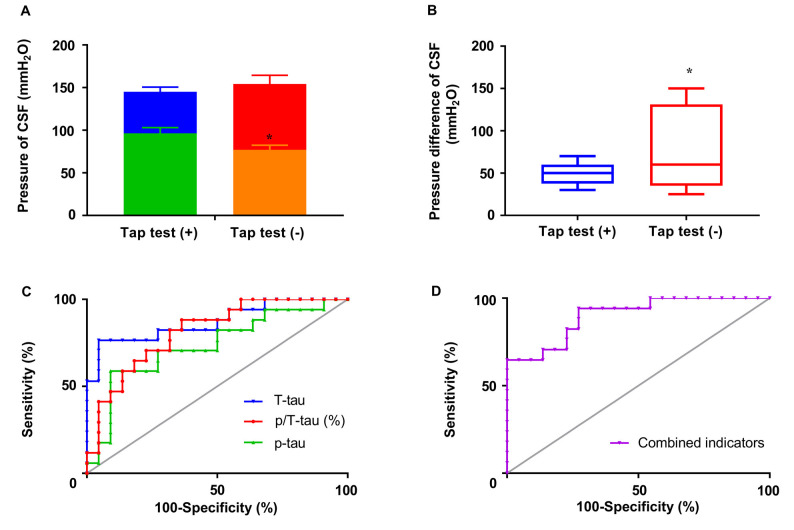
The responsiveness to the tap test and its correlated factors. **(A)** The differences in the initial and end pressure of CSF in patients with differences in tap test responsiveness. **(B)** The pressure difference in CSF in patients with differences in tap test responsiveness. **(C)** ROC curves of *T*-tau, ^181^*p*-tau, and ^181^*p*/*T*-tau levels in assessing tap test responsiveness. **(D)** ROC curves of combined indicators (−5.505 + 0.553 * percentage of p/*T*-tau − 1.586 * numbers of combined symptoms) in assessing tap test responsiveness (*p < 0.05 compared with the corresponding group. The data in **(B)** are presented as the medians with interquartile ranges).

In addition to combined symptoms, a correlation was also found between the end pressure or pressure difference of CSF and tap test responsiveness (*p* < 0.05), although no significant correlation was observed between tap test responsiveness and the initial pressure of CSF (Figures 5A,B). The initial pressure of CSF was 145.12 ± 22.49 mmH_2_O (*n* = 17) and 154.18 ± 47.73 mmH_2_O (*n* = 22) in the tap test (+) and (−) groups, respectively, which was similar. The end pressure was 96.35 ± 27.74 mmH_2_O (*n* = 17) in the tap test (+) group, which was significantly higher than that in the tap test (−) group (77.05 ± 25.24 mmH_2_O, *n* = 22; *p* < 0.05; Figure 5A). The Mann-Whitney U test was used to compare data for the pressure difference in CSF with a non normal distribution between the tap (+) and (−) groups. The pressure difference in CSF was 50.00 (47.50, 66.00) mmH_2_O in the tap test (+) group, which was lower than the value of 60.00 (33.75, 78.75) mmH_2_O recorded in the tap test (−) group (*p* < 0.01; Figure 5B). This finding suggests better brain tissue compliance in the patients who were tap test-positive.

We conducted a further binary logistic regression analysis to clarify the contribution of these related factors to tap test responsiveness. According to the results, *P* (tap test responsiveness) = 1/1 + e^∧^ − (−5.505 + 55.314 * ratio of *p*/*T*-tau − 1.586 * numbers of combined symptoms), suggesting a predictive value for a higher ratio of *p*/*T*-tau and fewer combined symptoms. Inferential combined indicators were calculated equal to −5.505 + 0.553* percentage of *p*/*T*-tau − 1.586 * numbers of combined symptoms.

The results of the actual levels or ratio of *p*-tau/*T*-tau and the combined indicators were further analyzed using ROC curves to determine the sensitivity, specificity, and area under the ROC curve (AUC). The combined indicators produced the highest AUC of 0.90, compared with *T*-tau (0.87), *p*-tau (0.63), and *p*/*T*-tau (0.82; Figures 5C,D). The cutoff value (−0.77) was selected and involved maximizing Youden’s index (0.67), of which the sensitivity and specificity were 94.12% and 72.73%, respectively.

## Discussion

Based on accumulating evidence, Aβ and tau are involved in the pathophysiology of several neurodegenerative diseases, and differences in their levels have been identified in different diseases (Riemenschneider et al., 2003; Ritchie et al., 2013; Jeppsson et al., 2013; Baiardi et al., 2018). Patients with iNPH consistently present with lower CSF T-tau or Aβ_1–42_ levels, and higher p-tau levels (Graff-Radford, 2014; Jingami et al., 2015a, b). Consistent with previous studies (Santangelo et al., 2017), our previous meta-analysis also showed the comparatively lower CSF Aβ_1–42_, T-tau, and p-tau levels in patients with NPH compared with patients with AD (Chen et al., 2017). However, the decreased levels of APP (Aβ_38_, Aβ_40_, Aβ_42_, sAPPα, and sAPPβ) and APP-derived proteins have no potential as indicators of shunt responsiveness (Jeppsson et al., 2016). In contrast, some patients with iNPH with co-existing neurodegenerative disorders presented increased CSF T-tau levels (Jingami et al., 2015b), although these patients might also benefit from a ventriculoperitoneal shunt, which also reduces the specific diagnostic value of tau and increases the difficulty of determining the diagnosis and prognosis (Craven et al., 2017). Unexpectedly, T-tau levels were also shown to be affected by changes in CSF hydrodynamics, while *p*-tau, levels especially ^181^p-tau levels, may more stably reflect pathological changes in the brain of patients with iNPH (Herukka et al., 2015). Therefore, we selected CSF T-tau, ^181^p-tau, and Aβ_1–42_ levels to assess their value in predicting shunt responsiveness based on tap test results. Simultaneously, other influencing factors were analyzed to identify better biomarkers or combined factors for the diagnosis and prognostic assessment of patients with iNPH.

The levels of CSF biomarkers were related to tap test responsiveness. In contrast to previous studies (Lim et al., 2014), Aβ1–42 did not affect tap responsiveness when AD was excluded as a comorbidity due to its effect on phonemic categorical naming and frontal inhibitory function. The levels of tau and its phosphorylated form were decreased in the CSF of patients with iNPH, especially in the tap test (+) group. The reduction in the levels of CSF proteins in patients with iNPH may result from the facilitated metabolic product drainage (Xie et al., 2013), dysfunction of CSF fluid dynamics (Herukka et al., 2015), interstitial fluid disorder in the glymphatic system (Iliff et al., 2012), and blockage of Aβ and tau protein outflow to the ventricular space induced by brain atrophy (Graff-Radford, 2014). However, the ratio of p/*T*-tau in the tap test (+) group was higher, suggesting that the magnitude of the reduction in *p*-tau levels is significantly lower than that of *T*-tau levels, resulting in a relatively higher ratio of p/*T*-tau. Moreover, the leve ratio of *p*-tau/Aβ1–42 and the responsiveness to the tap test were related to the number of combined symptoms in patients with iNPH. The number of combined symptoms was also affected by the prevalence of hypertension, initial pressure, and pressure difference in CSF. Alternatively, in addition to combined symptoms, a correlation was also observed between the end pressure or pressure difference of CSF and tap test responsiveness, which may suggest better brain tissue compliance in patients with tap test positivity. Collectively, a “concentric”-like relationship was observed between tap test responsiveness with the ratio of p/*T*-tau, pressure difference in CSF and numbers of combined symptoms, while the latter two factors were affected by hypertension and the initial or end pressure of CSF.

Further binary logistic regression analyses were conducted to clarify the contributions of these related factors to tap test responsiveness. The results showed that *P* (tap test responsiveness) = 1/1 + e^∧^ − (−5.505 + 55.314 * ratio of p/*T*-tau − 1.586 * numbers of combined symptoms). The inferential combined indicators were calculated as −5.505 + 0.553 * percentage of p/*T*-tau − 1.586 * numbers of combined symptoms. Further analysis of the ROC curve results showed that the combined indicators produced the highest AUC of 0.90, and the cutoff value was −0.77, which had a sensitivity and specificity of 94.12% and 72.73%, respectively. All of the results revealed the correlation between CSF biomarkers, clinical symptoms, and tap test responsiveness, which lays a research foundation for exploring the correlation as an index for evaluating clinical symptoms and the shunt response. We further calculated the cutoff value for combined indicators with the highest sensitivity and specificity using numbers of combined symptoms and p/*T*-tau in the judgment of tap test responsiveness, which is beneficial for the feasibility of a clinical application.

This study has some limitations. The number of patients in the subgroups of this study was low. Of course, a sex bias of the subjects exists in this study, and thus a larger sample is needed. This study only analyzed the predictive value of Aβ1–42, *T*-tau, and ^181^*p*-tau levels in patients with iNPH, although some discrepancy exists among different studies regarding the association between CSF markers and tap test responsiveness. For example, Aβ1–40 levels are also altered in patients with NPH (Jeppsson et al., 2013, 2016) and the Aβ1–42/Aβ1–40 ratio, a good predictor of the degree of changes in Aβ levels in brains of patients with AD (Janelidze et al., 2016; Baiardi et al., 2018), showed a higher diagnostic value for distinguishing iNPH from AD (Abu-Rumeileh et al., 2019). In fact, we only chose Aβ1–42 and not Aβ1–40 in our research based on the results of our previous meta-analysis of differentially altered CSF biomarkers in patients with iNPH (Chen et al., 2017). The setup of this study is also a potential limitation due to the ambiguous concentration gradient of biomarkers in the 2 × 15 ml CSF draw. The first milliliters of a CSF draw from patients with iNPH should be considered and analyzed in future verification studies, in which the difference in levels would perhaps be more pronounced, resulting in an improved diagnostic performance (Graff-Radford, 2014; Jingami et al., 2019). Moreover, the intrinsic molecular mechanism of CSF biomarkers in the progression of iNPH still requires further exploration.

## Conclusions

The analysis of CSF biomarkers from patients with iNPH showed decreased levels of tau and its phosphorylated form, especially in the tap test (+) group, although the ratio of p/*T*-tau was relatively higher. Alternatively, the responsiveness to the tap test was related to the number of combined symptoms, end pressure, and pressure difference. Further analysis showed that combined indicators (−5.505 + 0.553 * percentage of p/*T*-tau − 1.586 * numbers of combined symptoms) yielded the highest area under the ROC curve of 0.90. The cutoff value (−0.77) was determined by maximizing Youden’s Index (0.67), of which the sensitivity and specificity were 94.12% and 72.73%, respectively.

## Data Availability Statement

The raw data supporting the conclusions of this article will be made available by the authors, without undue reservation.

## Ethics Statement

The studies involving human participants were reviewed and approved by Ethics Committee of Aviation General Hospital. The patients/participants provided their written informed consent to participate in this study. Written informed consent was obtained from the individual(s) for the publication of any potentially identifiable images or data included in this article.

## Author Contributions

Conceptualization: RH and YX. Methodology and supervision: YL. Software, data curation, writing—original draft preparation, and project administration: RH. Validation: CL and XL. Formal analysis: JZhu. Investigation: JZha, LW, ZS, JL, SK, CY, NL, LL, JS, QY, and YW. Resources: YZ. Writing—review and editing: YX. All authors contributed to the article and approved the submitted version.

## Conflict of Interest

The authors declare that the research was conducted in the absence of any commercial or financial relationships that could be construed as a potential conflict of interest.
